# Feasibility and Acceptability of Game-Based Cortical Priming and Functional Lower Limb Training in a Remotely Supervised Home Setting for Chronic Stroke: A Case Series

**DOI:** 10.3389/fresc.2022.775496

**Published:** 2022-02-22

**Authors:** Hyosok Lim, Nicholas Marjanovic, Cristian Luciano, Sangeetha Madhavan

**Affiliations:** ^1^Brain Plasticity Laboratory, Department of Physical Therapy, University of Illinois at Chicago, Chicago, IL, United States; ^2^Graduate Program in Rehabilitation Sciences, College of Applied Health Sciences, University of Illinois at Chicago, Chicago, IL, United States; ^3^Mixed Reality Laboratory, Department of Biomedical Engineering, University of Illinois at Chicago, Chicago, IL, United States

**Keywords:** stroke, gamification, cortical priming, walking, telerehabilitation

## Abstract

**Background:**

Movement-based priming has been increasingly investigated to accelerate the effects of subsequent motor training. The feasibility and acceptability of this approach at home has not been studied. We developed a game-based priming system (DIG-I-PRIME^TM^) that engages the user in repeated ankle movements using serious games. We aimed to determine the feasibility, acceptability, and preliminary motor benefits of an 8-week remotely supervised telerehabilitation program utilizing game-based movement priming combined with functional lower limb motor training in chronic stroke survivors.

**Methods:**

Three individuals with stroke participated in a telerehabilitation program consisting of 20-min movement-based priming using the DIG-I-PRIME^TM^ system followed by 30-min of lower limb motor training focusing on strength and balance. We evaluated feasibility using reported adverse events and compliance, and acceptability by assessing participant perception of the game-based training. Motor gains were assessed using the 10-m walk test and Functional Gait Assessment.

**Results:**

All participants completed 24 remotely supervised training sessions without any adverse events. Participants reported high acceptability of the DIG-I-PRIME^TM^ system, reflected by high scores on satisfaction, enjoyment, user-friendliness, and challenge aspects of the system. Participants reported overall satisfaction with our program. Post-training changes in the 10-m walk test (0.10–0.31 m/s) and Functional Gait Assessment (4–7 points) exceeded the minimal clinically important difference.

**Conclusion:**

Our results indicate that a remotely supervised game-based priming and functional lower limb exercise program is feasible and acceptable for stroke survivors to perform at home. Also, improved walking provides preliminary evidence of game-based priming to be beneficial as a telerehabilitation strategy for stroke motor recovery.

## Introduction

Post-stroke individuals experience barriers that limit access to necessary physical or occupational therapy that is critical to regain and maintain motor function. Some of these barriers include health care costs, time to travel, lack of transportation, lack of available resources in hospitals/clinics, lack of caregiver support, etc., and these barriers have only become greater in the era of COVID-19 pandemic (1). Telerehabilitation, defined as the remote delivery of rehabilitation services *via* communication technologies, is being explored as a viable option for stroke survivors to safely and easily access high quality training at home (2). The effectiveness of post-stroke telerehabilitation has been established (3). Studies have reported improvements in lower limb function and balance after telerehabilitation to be comparable to clinic-based rehabilitation in stroke survivors (4, 5). While comparable improvements in a remotely supervised setting is commendable, there remains a compelling need to maximize the benefits of standard-of-care motor rehabilitation.

Majority of individuals after stroke continue to experience impairment in lower limb motor function resulting in reduced walking speeds and abnormal gait symmetry (6). Compromised walking affects an individual's community engagement, increases risk of falls, and lowers quality of life (7). The primary goal of majority of stroke survivors is to improve walking ability (8), however, this need is not met by current approaches to gait training (9). There remains an urgent need to maximize the effectiveness of walking interventions to optimize functional recovery after stroke.

Cortical priming, an adjuvant approach to enhance brain plasticity during or prior to motor training, has been explored as a promising strategy to maximize outcomes of motor training (10). Movement-based priming is one such cortical priming approach that utilizes specific movement paradigms to activate the brain (11). Greater corticomotor excitability and enhanced motor performance in the upper limb has been reported when motor training is preceded by movement-based priming in individuals with stroke compared to training alone (12–14). Despite its enhanced benefits, movement-based priming has not been used to prime post-stroke gait rehabilitation. In addition, the utility of movement-based priming as a telerehabilitation approach has not been explored.

**Figure 1:**
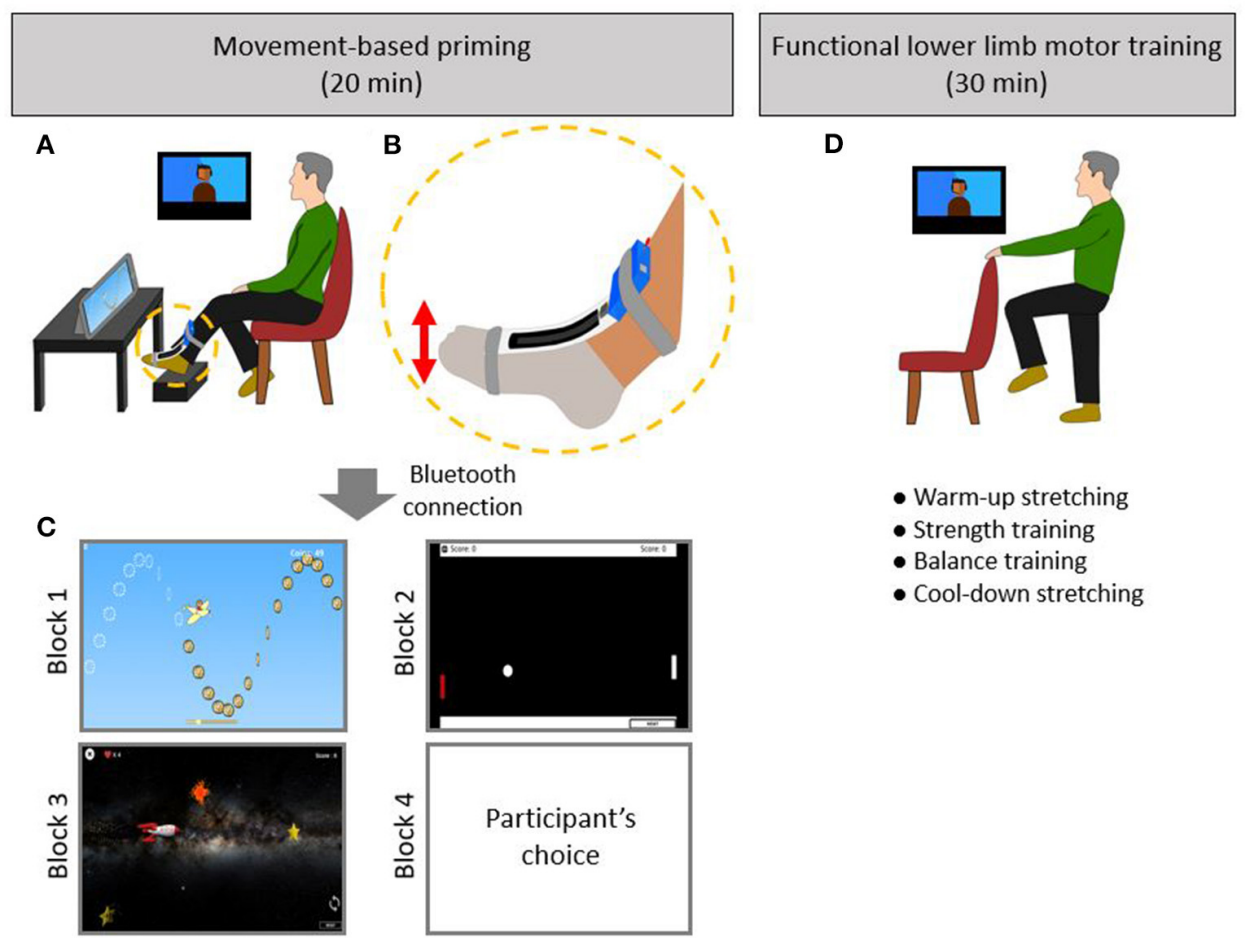
**(A)** DIG-I-PRIME^TM^ system setting with remote supervision *via* videoconference for 20-min of movement-based priming. **(B)** DIG-I-PRIME^TM^ foot piece consists of a microcontroller (blue) and a bend sensor (black). The bend sensor was centered on the dorsum of the paretic foot while the microcontroller faced the participant. The DIG-I-PRIME^TM^ foot piece was supported by Velcro straps (dark gray). The DIG-I-PRIME^TM^ foot piece wirelessly transmitted information regarding foot orientation in the sagittal plane during ankle dorsiflexion and plantarflexion movements to the tablet *via* Bluetooth connection. **(C)** Four blocks of video games (5-min each) were played using the tablet placed in front of the participants. Three games, which included Treasure Hunt (Block 1), Ping-Pong (Block 2), and Shooting Stars (Block 3), were played. The participant chose on of these three games for Block 4. **(D)** Following 20-min of priming, participants performed 30 min of functional lower limb motor training under remotely supervision.

To overcome this critical gap in current stroke rehabilitation practice, we developed a game-based technology for movement-based priming that can be easily translated to a home-based setting to provide an engaging and motivating priming experience. The DIG-I-PRIME™ system (developed by the research team SM and CL) incorporates a bend sensor strapped to the ankle that communicates wirelessly to a digital application and engages the user in games utilizing repeated skilled ankle movements (Figure 1). The DIG-I-PRIME™ system is an affordable and accessible option for telerehabilitation as the hardware and software is built using off-the-shelf systems and is cost effective (below $500), compared to other currently available gaming systems.

Interactive gaming has been increasingly incorporated within stroke motor rehabilitation in the laboratory and clinical settings, and has shown to demonstrate greater motor improvements and compliance compared to conventional therapies (15–17). Game playing increases user motivation and engagement which are important factors for compliance and adherence to maximize benefits of motor training (18, 19). To the best of our knowledge, the present study is the first to integrate gamification with movement-based priming in stroke survivors, especially in a remotely supervised setting.

We recently demonstrated a significant priming effect (upregulated corticomotor excitability in the ipsilesional motor cortex) in chronic stroke survivors after 20 min of repeated movements of the paretic ankle during serious game playing using the DIG-I-PRIME^TM^ system in the laboratory (20). This pilot study established the cortical priming effect of game-based movements. In the current case series, our objective was to explore whether game-based priming can be translated to the home setting and expand the scope of priming to include motor training for functional walking recovery. Specifically, this study aimed to determine the feasibility and acceptability of an 8-week remotely supervised game-based movement priming combined with lower limb motor training in chronic stroke survivors. The secondary aim was to determine the preliminary motor gains of the priming and telerehabilitation program on walking outcomes.

## Methods

### Study Overview

In this single-group feasibility study, individuals with stroke participated in 24 sessions of a remotely supervised telerehabilitation program over 8 weeks. Each session consisted of 20 min of game-based movement priming using DIG-I-PRIME^TM^ followed by 30 min of functional lower limb motor training. Training sessions were performed at home with remote supervision by a research personnel using videoconferencing. Participants were required to complete in-person lab visits for assessments at baseline, mid-training (4-week), and post-training (8-week).

### Participants

Three individuals with chronic stroke participated in this case series. Inclusion criteria included diagnosis of a single mono-hemispheric stroke for more than 6 months, age between 40 and 80 years, minimum of 5 degrees of active ankle dorsiflexion necessary for movement-based priming, ability to walk independently for at least 5 min with or without an assistive device, and ability to sit unsupported for 30 s. In addition, participants were expected to have access to the internet and own communicating devices such as a smartphone, tablet, or laptop with an on-board camera to enable monitoring *via* videoconference. The study excluded stroke participants with lesions pertaining to the brainstem or cerebellum, lower limb contractures, significant cognitive impairment (Mini Mental State Examination <21), chronic cardiorespiratory or metabolic diseases, and severe osteoporosis. The study was approved by the Institutional Review Board at the University of Illinois at Chicago.

### Participant Familiarization to Priming and Training Protocol

Screening and baseline assessments were followed by an additional lab visit to familiarize the participant to the training program. This visit trained the participant on how to operate the DIG-I-PRIME^TM^ system, perform functional lower limb exercises, and access videoconferencing independently. A standardized aptitude checklist was used to ensure that each participant reached a minimum level of competency in all areas to ensure safety and proficiency during performance of study protocol at home. If the participant did not pass the checklist the first time, the research personnel repeated the training until competency was achieved. We found that participants required a maximum of two demonstrations before achieving competence. Participants were then dispatched with the necessary equipment for priming and lower limb exercises such as the DIG-I-PRIME^TM^ system, Therabands and a foam block to raise the heel during priming. We also included user manuals for the DIG-I-PRIME^TM^ system and for the exercise protocol, and a training checklist. Before initiating training, we conducted a remote session to ensure that the participant was able to videoconference from home with the necessary equipment such as a chair or bed, and were able to place their personal phone/tablet/laptop in a position that enabled the research personnel to view the participant fully for remote supervision.

### Remote Supervision

Participants were remotely supervised by skilled research personnel to ensure proper and safe performance of the protocol. Remote supervision was conducted *via* HIPAA-compliant videoconferencing using the Doxy.me telemedicine platform (https://doxy.me). Before each session, the participants entered the video chat room using their personal electronic device (tablet or laptop) by clicking on a link that was provided by the research personnel. Then, the electronic device was placed in a direction and location which allowed a clear view of participant performance. During remote supervision, research personnel helped the participant progress through the sequence by cueing them to the next exercise, provided feedback, and answered questions as necessary.

### Movement-Based Priming

Participants performed 20 min of ankle movements using the DIG-I-PRIME^TM^ system while seated on a chair, resting the heel of their paretic leg on a high-density non-slip foam block to allow full range of ankle motion. Participants performed three simple and engaging games (Treasure Hunt, Ping-Pong, and Shooting Stars) using ankle movements. At the beginning of the session, the games were calibrated to include movements that corresponded to 80% of each participant's maximum range of motion. Detailed game information is provided in Lim et al. (20). The main objective of each game was to accurately reach the targets (coins, ping-pong ball, or stars) utilizing continuous ankle movements. Participants engaged in four blocks of serious game playing, with each block lasting for 5 min with 1 min rest in between. Treasure Hunt, Ping-Pong, and Shooting Stars were played for the first three blocks. The fourth block consisted of the participant's choice of the three games. Participants were provided a score based on the number of targets collected out of total number of targets (Treasure Hunt and Shooting Stars) or number of Ping-Pong games won against the AI at the end of each trial. To progress the challenge levels, speed of the targets increased by 10% in the next trial whenever the participant achieved 80% of the target accuracy (Treasure Hunt and Shooting Stars) or won five Ping-Pong games with no more than one loss. Verbal instructions were provided by the supervising research personnel not to lift the heel off from the foam block during game-playing to isolate the movements to the ankle.

### Functional Lower Limb Motor Training

After the movement-based priming, participants performed 30 min of functional lower limb motor training which included stretching, strength, and balance exercises. The motor training protocol used in this study was adapted with modifications from previous studies which have successfully demonstrated the feasibility of these exercises in stroke survivors without compromise to participant safety (5, 21). The training consisted of 2-min seated stretching as a warm-up (1 set of 10 repetitions for ankle dorsiflexors, ankle plantarflexors, knee flexors, knee extensors, hip abductors, and hip adductors), 12-min strength exercises using the TheraBand (2 to 3 sets of 10 repetitions for hip flexion, hip extension, hip abduction, hip adduction, knee flexion, and knee extension), 12-min balance exercises (2–3 sets of 10 repetitions for seated reaching, supine bridging, supine heel slides, and supine to sitting), and 4-min supine/seated stretching as a cool-down (30 s each for ankle dorsiflexors, ankle plantarflexors, knee flexors, knee extensors, hip flexors, and hip extensors). Participant's progress was remotely evaluated weekly by a physical therapist using standardized criteria. Based on the weekly evaluation, the intensity of exercises was increased by progressing to a TheraBand with greater resistance or progressing to standing exercises for strength or proceeding to standing balance including heel raises, sit to stand, spot marching, and dynamic standing. Verbal instructions were given to the participants *via* videoconference to ensure safety (e.g., clear the exercise area, provide rest whenever necessary, verbal warning of potential risks) and proper performance of the exercises at home. List of functional lower limb exercises is provided in the Supplementary Materials.

### Outcome Measures

We assessed *feasibility* by examining the number of adverse events and participant compliance at the end of 24 sessions.

- Adverse events: The number of adverse events related to the DIG-I-PRIME^TM^ and lower limb motor training was recorded during the remotely supervised sessions. An adverse event was defined as an event that results in study-related discomfort such as pain, injury, or fall.
- Compliance: The number of sessions completed by each participant was recorded. Each session was considered complete only if all the procedures within a session was completed by the participant.

We assessed participant *acceptability* of the DIG-I-PRIME^TM^ system using a structured feedback form with 20 Likert-scale type questions, and the telerehabilitation program was evaluated by three open-ended questions at the end of training.

- Acceptability: The Likert-scale type questions were subcategorized into four sections: enjoyment, user-friendliness, challenge, and satisfaction. Participants were asked to rate each question on a scale from 1 to 5 (1 = strongly disagree, 2 = disagree, 3 = somewhat agree, 4 = agree, and 5 = strongly agree). During the analyses, the rating scale was reversed for the questions containing negative item. For example, the negative item, “I felt pain during the training,” scored 1 (strongly disagree) was reversed to score of 5. Then, each category was scored using an average of the reversed scores for each participant (out of 5) with 5 indicating positive feedback. Participants filled out three open-ended questions which include strengths and weaknesses of the telerehabilitation program.

We assessed *motor gains* as a secondary outcome by evaluating walking function at baseline, mid-testing, and post-testing. All evaluations were conducted by one assessor.

- Walking speed: Three trials of the 10-m walk tests (10MWT) were averaged. Ten MWT has shown excellent reliability and validity to assess walking speed in stroke survivors (22). Participants walked at their fastest speed on a 10-m walkway.
- Dynamic balance: Functional Gait Assessment (FGA) was used to assess dynamic balance during different walking tasks. FGA has shown sufficient reliability and validity to assess dynamic balance in stroke survivors (23). FGA contains 10 different walking tasks which are scored on a 4-level ordinal scale (0–3) for each item with the total score ranging from 0 to 30. Lower score indicates poor dynamic balance.

## Results

### Participants

Three individuals with chronic stroke (mean 7.9 ± 4.4 years) enrolled and completed the study. Participant 1 (P1) was a 55-year-old Hispanic male with hemorrhagic stroke with unknown lesion location presenting with right-sided hemiparesis. He scored 20 out of 34 on the Fugl-Meyer lower extremity assessment (FMLE) and 18/26 degrees for active dorsiflexion/plantarflexion range of motion in the paretic side during the initial evaluation. P1 used assistance from a walker for his daily activities. Participant 2 (P2) was a 48-year-old African American female with ischemic stroke in the internal capsule. She presented left-sided hemiparesis with a FMLE score of 15 and active dorsiflexion/plantarflexion range of motion of 6/16 degrees. She used an electronic wheelchair for her daily activities, however, was able to perform walking tests without assistance. Participant 3 (P3) was a 69-year-old Caucasian male with hemorrhagic stroke in the basal ganglia. He presented with left-sided hemiparesis with FMLE score of 25 and active dorsiflexion/plantarflexion range of motion of 18/14 degrees. P3 did not use any walking aids during daily activities and demonstrated high independence during walking.

### Feasibility

A total of 72 sessions was completed by all three participants (24 sessions for each participant, 100% compliance). Overall remote supervision was successful except in one session where P1 was remotely supervised using only audio during priming instead of videoconference due to the unstable internet connection at the participant's home. The supervising research personnel were not able to visually observe the completion of the priming, however, were able to confirm the completion based on the game data stored in the tablet. Training schedules were maintained flexible for each participant and the location of training was preferred but not restricted to participant's home. For example, P3 was able to carry all necessary training equipment to his vacation location and completed three remotely supervised training sessions on vacation. The training location used for these three sessions was observed and pre-approved by the research personnel through videoconference prior to the start of the session. No adverse events were noted by the participants or research personnel during the training period.

### Acceptability

Participant acceptability of the DIG-I-PRIME^TM^ system (Table 1) demonstrated highest score for “satisfaction” from all participants (range 4.4–5.0; average 4.8), followed by “enjoyment” (range 4.0–5.0; average 4.4), “user-friendliness” (range 3.6–4.6; average 4.1), and “challenge” (range 3.6–4.2; average 3.8). Table 2 presents the three open-ended questions that assessed participant acceptability of the telerehabilitation program. P1 was satisfied with the variety of games, however, suggested the need of the DIG-I-PRIME^TM^ foot piece to be more convenient for attachment using only one hand. P2 reported the interaction with the research personnel *via* videoconference to be motivational and helpful. P2 also suggested a wider range of challenge levels for the games/exercises, while P3 was satisfied with the different levels of challenge provided. P3 was also satisfied with the accessibility of the program due to the portability of the DIG-I-PRIME^TM^ system and exercise equipment. However, P3 reported the difficulty of plugging the charger into the port on the DIG-I-PRIME^TM^ foot piece which is consistent with the comment from P1 suggesting the foot piece to be designed better for those using only one hand.

**Table 1:** Participant acceptability of the DIG-I-PRIME^TM^ system.

**Questions rating DIG-I-PRIME^**TM**^**	**Score (1–5)**		
	**P1**	**P2**	**P3**
**Enjoyment**			
I got bored with the games*	3	2	5
I felt stressed during the training*	4	4	5
I enjoyed the training	3	5	5
I felt pain during the training*	5	5	5
I felt depressed during or after the training*	5	5	5
**Average**	**4.0**	**4.2**	**5.0**
**User-friendliness**			
The instructions for training were easy to understand	4	5	5
The device was user-friendly	5	5	5
The games were hard to see using the tablet*	3	5	5
I had trouble putting on and taking off the ankle device*	3	3	3
I had trouble operating the gaming software on the tablet*	3	3	5
**Average**	**3.6**	**4.2**	**4.6**
**Challenge**			
This training challenged me	3	5	3
This training was too difficult*	3	3	5
This training was too easy*	3	4	3
I felt tired during or after training*	5	3	5
The pace of training was just right	3	4	5
**Average**	**3.4**	**3.8**	**4.2**
**Satisfaction**			
This training was meaningful to me	5	5	5
My function has improved	4	5	5
The games were appropriate for my age	5	5	5
If this device is available, I would likely use it at home	3	5	5
I am satisfied with the progress I made using the device	5	5	5
**Average**	**4.4**	**5.0**	**5.0**

* *Indicates negative (reverse) items. An original rating scale from 1 to 5 (1, strongly disagree; 2, disagree; 3, somewhat agree; 4, agree; 5, strongly agree) was reversed for the indicated negative items presented above. Hence, the scores presented in the table ranges from 1 to 5, 1 being negative feedback and 5 being positive feedback*.

**Table 2:** Participant acceptability of the telerehabilitation protocol.

**Open-ended questions**	**Response**
What would you change about the game/device or exercise training?	P1 “device could be designed to attach on my foot more easily”
	P2 “make different challenge levels available for games and exercises”
	P3 “charging port should be designed easier to plug in”
What would you change about other aspects of the program? (length of training, trainer feedback/instruction etc.)	P1 “nothing” P2 “nothing” P3 “length of training could have been longer”
What did you like best about the program?	P1 “enjoyed different games”
	P2 “interacting with the therapist through videoconference was encouraging” and “enjoyed different games”
	P3 “can be trained anywhere” and “enjoying and challenging with different levels of game/training”

### Walking Outcomes

Table 3 presents walking outcomes at baseline, mid-testing, and post-testing. P1 and P2 increased fastest walking speed at mid-testing (45 and 4%, respectively) while P3 showed no change. All participants demonstrated an increase in fastest walking speed at the post-assessment (P1: 46%, P2: 16%, and P3: 6%). For FGA, participants showed increased scores at mid-testing (P1: 25%, P2: 15%, P3: 7%) and demonstrated greater improvement at the end of training (P1: 42%, P2: 31%, P3: 47%).

**Table 3:** Clinical outcomes.

	**Baseline**	**Mid-testing**	**Post-testing**
**10MWT (fastest speed, m/s)**			
P1	0.68	0.98*	0.99*
P2	0.94	0.97	1.09*
P3	1.72	1.73	1.82*
**FGA (out of 30)**			
P1	12	15	17*
P2	13	15	17*
P3	15	16	22*

*10MWT, 10-meter walk test; FGA, functional gait assessment*.

* *indicates changes from baseline exceeding minimal clinically important differences (MCID)*.

## Discussion

Our objective for this study was to determine the feasibility, acceptability, and preliminary motor gains of a 24-session remotely supervised telerehabilitation program utilizing a novel game-based device for cortical priming followed by functional lower limb motor training in chronic stroke survivors. Three stroke participants enrolled in this study were able to complete 24 training sessions without adverse events. Participants were overall satisfied with the telerehabilitation program, especially with the enjoyability of the games, user-friendliness of the device, feasibility and accessibility of the program, and interaction with the research personnel through videoconference. All participants showed mild to modest increases in walking speed and dynamic balance at the end of the telerehabilitation program.

### Feasibility and Acceptability

Participants demonstrated 100% compliance without any adverse events during the remotely supervised telerehabilitation program. This is meaningful as none of our participants reported to have a caregiver at home and one of our participants demonstrated severe motor impairment of the paretic limb indicating our program to be feasible and safe for stroke survivors, even with limited functional ability, to perform without any assistance. Similar to our results, other walking related telerehabilitation studies have demonstrated high compliance (80–100%) during 20 sessions (over 4–8 weeks) of home-based training in stroke survivors (4, 24). The 100% compliance achieved in our study may be due to relatively short duration of training (8 weeks). Repetitive training on the same protocol for an extended period can limit adherence. Although larger doses of telerehabilitation have been reported to demonstrate greater motor benefits (25), incorporating cortical priming techniques to telerehabilitation may provide motor benefits without requiring longer durations of training. The presence of research personnel *via* videoconference for every session may have contributed to our high compliance. Regular interaction between health professional and patients has shown to enhanced motivation and increase compliance during telerehabilitation (26, 27). This is evident in a recent telerehabilitation study demonstrating 22% decrease in compliance after reducing the number of remotely supervised session in stroke survivors (28).

Participant acceptability of the priming with the DIG-I-PRIME^TM^ system was rated as highly positive for satisfaction and enjoyment after 8-weeks of telerehabilitation. Satisfaction and enjoyment during therapy have shown to enhance participant engagement and facilitate greater motor gains in stroke survivors (29). Although the “user-friendliness” category was rated positive (4.1 out of 5), all participants commonly rated ‘moderate' on the difficulties of attaching or detaching the DIG-I-PRIME^TM^ foot piece, which was also mentioned by P1 in the open-ended questions. As perceived ease of use is identified as an important factor for older adults to adopt interventions utilizing gaming technology (30), our future aim is to upgrade the design of the foot piece to allow easy operation for those with limited hand function. Response to the question about “perceived challenge” differed between participants possibly due to the different impairment levels in the paretic limb. At baseline, each participant was identified to have mild, moderate, and severe lower limb impairment reflected by the FMLE score of 25 for P3, 20 for P1, and 15 for P2, respectively. P1 and P3 who demonstrated mild to moderate impairment in the paretic lower limb did not find the games to be challenging while P2 who had severe impairment found it challenging. We will consider providing tailored initial challenge levels for each game based on participant's functional capacity.

### Walking Outcomes

Twenty-four sessions of telerehabilitation resulted in clinically relevant improved walking speed in our participants. At post-testing, change in walking speed ranged from 0.10 to 0.31 m/s which is equal or greater than the minimal clinically important difference (MCID) for walking speed (0.10 m/s) (31). P1 improved walking speed at mid-testing, while P2 and P3 increased walking speed at post-testing. Faster improvements in P1 may be due to their lower walking speed at baseline, while those with higher initial walking speed (P2 and P3) needed to complete the 8-week program to show changes. This is consistent with previous evidence demonstrating the importance of high-dosage exercise to improve motor function in mildly impaired chronic stroke survivors (32). Considering that at least 36 treadmill training sessions were reported to be necessary to achieve an increase in walking speed exceeding the MCID (33), our participants achieved the meaningful walking speed changes within a shorter period (12–24 sessions) which is possibly due to the movement-based priming conducted prior to the functional lower limb motor training. The priming effect may have facilitated neuroplasticity helping accelerate functional gains for the subsequent motor training. Enhanced benefits of motor training preceded by priming compared to motor training alone has been previously reported (12–14). However, our findings cannot confirm efficacy due to the case series nature of this study and lack of control.

The FGA changes ranging from 4 to 7 at the end of training are considered clinically meaningful according to the established MCID of 4 points for the FGA scale (34). The strength training focusing on the paretic lower limb and balance training components may have improved dynamic balance in our participants contributing to greater stability during walking (35). Interestingly, P3 showed greater changes in FGA (change of 7) at the end of training despite the high baseline FGA score compared to others (P1 and P2).

### Limitations and Future Studies

The present study has several limitations. First, the case series nature of this paper limits the rigor of our findings. Our intention for this study was to determine the feasibility and acceptability of the DIG-I-PRIME^TM^ system and functional lower limb motor training for stroke survivors in a home setting before progressing to a clinical trial. Second, we did not include a control group. Although, our telerehabilitation program demonstrated clinically relevant improvements, it is still unclear if the improvements are derived from the functional lower limb motor training facilitated by the priming effect or functional lower limb motor training alone. A future larger trial including a control group without movement-based priming is warranted. Third, we noticed that the design of the DIG-I-PRIME^TM^ foot piece was less efficient for stroke survivors to attach on the foot. The idea of utilizing Velcro straps to attach the device was to accommodate participants with different foot sizes, however, this seemed to be challenging for some participants with high impairment of the paretic hand. Our goal is to modify the device attachment by perhaps utilizing elastic straps that “slide-on/off” which may allow easy application and proper orientation of the device on the foot using only one hand. Fourth, our participant acceptability questionnaires were limited to mainly assess the acceptability of the DIG-I-PRIME^TM^ system. Although, some positive participant responses were received for the remote supervision from the open-ended questions, addition of a questionnaire that collects data regarding remote supervision and other aspects of telerehabilitation may be beneficial. Fifth, the DIG-I-PRIME^TM^ was limited to utilizing only dorsiflexion and plantarflexion movements during game playing. Allowing multiple degrees of freedom of ankle movement such as inversion or eversion may involve more muscle representations that contribute to balance during walking.

## Conclusion

In this case series, we have demonstrated the feasibility, acceptability, and potential motor benefits of an 8-week telerehabilitation program utilizing game-based movement priming followed by functional lower limb motor training with remote supervision in chronic stroke survivors. Our telerehabilitation protocol provides a viable option for stroke survivors to easily implement engaging motor training at home utilizing a movement-based priming system that has the potential to enhance walking post-stroke. In addition, the functional lower limb motor training protocol presented in this study was observed to be safe and feasible for performing independently at home, even for those with a high impairment level. This is the first study to demonstrate feasibility and potential ambulatory changes in chronic stroke survivors utilizing a movement-based priming approach targeting the lower limb in a remotely supervised home setting. Future larger controlled studies are needed to establish the effectiveness of game-based priming and telerehabilitation to optimize lower limb motor recovery in chronic stroke survivors.

## Data Availability Statement

The raw data supporting the findings of this article will be available from the corresponding author upon reasonable request.

## Ethics Statement

The studies involving human participants were reviewed and approved by Institutional Review Board at University of Illinois at Chicago. The patients/participants provided their written informed consent to participate in this study.

## Author Contributions

CL and SM conceptualized the work and developed the gaming system used in this study. HL, CL, and SM developed the study design. HL conducted the data collection and wrote the first draft of the manuscript. NM maintained the gaming system. HL and SM analyzed, interpreted the data, and contributed to manuscript revision. All authors read and approved the submitted version.

## Funding

This study was supported by the Chancellor's Innovation Fund through University of Illinois at Chicago's Proof of Concept Award.

## Conflict of Interest

The authors declare that the research was conducted in the absence of any commercial or financial relationships that could be construed as a potential conflict of interest.

## Publisher's Note

All claims expressed in this article are solely those of the authors and do not necessarily represent those of their affiliated organizations, or those of the publisher, the editors and the reviewers. Any product that may be evaluated in this article, or claim that may be made by its manufacturer, is not guaranteed or endorsed by the publisher.
