# Supplementary material for: Feasibility and Acceptability of Game-Based Cortical Priming and Functional Lower Limb Training in a Remotely Supervised Home Setting for Chronic Stroke: A Case Series
Source: Front Rehabil Sci. 2022 Feb 22;3:775496. doi: 10.3389/fresc.2022.775496 (PMC9397891; doi:10.3389/fresc.2022.775496)
Supplement: Supplementary file 1 [file Table_1.docx]

***Supplementary material***

- Functional lower limb motor training

|  | **Focus** | **Type** | **Level** | | **Progression** |
| --- | --- | --- | --- | --- | --- |
| 1 | **Warm-up Stretching (Seated)**  - Total duration: 2 min  - 1 set of 10 repetitions each | Ankle Dorsiflexors-Plantarflexors | - | | - |
|  |  | Leg Kicks  (Knee Flexors-Extensors) | | - | - |
|  |  | Sitting Butterfly (Hip Abductors-Adductors) | - | | - |
| 2 | **Strengthening**  - Total duration: 12 min  - 2 to 3 sets of 10 repetitions each  - Using TheraBand | Hip Flexion | 1 (sitting) / 2 (standing) | | Different resistance levels of TheraBand |
|  |  | Hip Extension |  |  |  |
|  |  | Hip Abduction |  |  |  |
|  |  | Hip Adduction |  |  |  |
|  |  | Knee Flexion |  |  |  |
|  |  | Knee Extension |  |  |  |
| 3 | **Balance**  - Total duration: 12 min  - 2 to 3 sets of 10 repetitions each | Seated Reaching | 1 | | One hand to no hand support |
|  |  | Supine Bridging | 1 | | Longer duration of hold (3, 5, 10 seconds) |
|  |  | Supine Heel Slides | 1 | | Slide against bed surface, shin, and air |
|  |  | Supine to Sitting | 1 | | Greater repetitions (10, 12, 15 times) |
|  |  | Sit to Stand | 2 | | No hand support |
|  |  | Heel Raises | 2 | | No hand support |
|  |  | Spot Marching | 2 | | No hand support |
|  |  | Dynamic Standing | 2 | | No hand support |
| 4 | **Cool-down Stretching**  - Total duration: 4 min  - 30 seconds each | Supine Quadriceps (Hip Flexors & Knee Extensors) | - | | - |
|  |  | Seated Hamstrings (Hip Extensors & Knee Flexors) | - | | - |
|  |  | Ankle Dorsiflexors | - | | - |
|  |  | Ankle Plantarflexors | - | | - |
